# Risk factors for carnitine deficiency in critically ill adults: A descriptive cross‐sectional study

**DOI:** 10.1002/jpen.70044

**Published:** 2026-01-07

**Authors:** Jennifer Gordon, Rebecca Brody, Hamed Samavat, Mireille Hamdan, Alexander L. Colonna, Jason B. Young, Laura D. Byham‐Gray

**Affiliations:** ^1^ Department of Clinical and Preventive Nutrition Sciences School of Health Professions Newark New Jersey USA; ^2^ University of Utah Hospital Salt Lake City Utah USA; ^3^ Mayo Clinic Jacksonville Florida USA

**Keywords:** carnitine deficiency, critical care, critical illness, L‐carnitine

## Abstract

**Background:**

Critical illness is a risk factor for carnitine deficiency. Carnitine deficiency may result in serious medical complications and poor clinical outcomes. This study aimed to identify the prevalence and potential predictors of carnitine deficiency.

**Methods:**

This was a descriptive cross‐sectional study conducted in a convenience sample of 144 critically ill adults admitted to a surgical or cardiovascular intensive care unit at the University of Utah Hospital, with serum carnitine levels measured from January 1, 2022, to December 31, 2024. Binary and multivariable logistic regression models were constructed to explore potential predictors of carnitine deficiency, which was defined as a free serum carnitine level <36 µmol/L.

**Results:**

The mean age of patients was 56.5 years; 41.7% of the sample had carnitine deficiency. For each unit decrease in body mass index, the odds of developing carnitine deficiency increased by 5% compared with maintaining normal free carnitine status (odds ratio [OR], 0.95; 95% confidence interval [CI], 0.9–0.99). Those prescribed continuous renal replacement therapy were 2.43 times more likely to develop carnitine deficiency than those not on continuous renal replacement therapy (OR, 2.43; 95% CI, 1.12–5.25).

**Conclusion:**

A high proportion of our study population had carnitine deficiency. Continuous renal replacement therapy and a lower body mass index were significant predictors in the multivariable model. Female sex and a history of chronic kidney disease may warrant further exploration based on significance in the univariable analysis.

## INTRODUCTION

Critical illness places patients at risk for L‐carnitine deficiency (hereafter referred to as carnitine) because of protein catabolism^1^ and possible related treatments including dialysis,^2^ parenteral nutrition,^3^ and medications such as valproic acid.^4^ In organs such as the heart and skeletal muscle, inadequate carnitine to transport long‐chain fatty acids intracellularly limits this key energy source.^1^ If beta‐oxidation cannot occur, muscles must quickly break down their glycogen stores, thereby diminishing the energy supply.^5^ Inadequate carnitine may result in complications such as hypoglycemia, elevated triglyceride levels, weakness, cardiac dysfunction,^6^ and mitochondrial dysfunction.^7^ A progression to multiorgan failure^7^ and, ultimately, poor clinical outcomes have been associated with mitochondrial dysfunction.^8^, ^9^ Other carnitine deficiency symptoms include fatigue,^10^ elevated ammonia and/or lactate levels,^2^ muscle wasting, and fatty liver (as evidenced by liver enzyme elevations).^11^ In a recent 2025 editorial review, Robert et al emphasized the lack of robust trials studying carnitine deficiency and its impact on critically ill patients.^12^ There is a need for research on nutrients that may help combat the mitochondrial dysfunction associated with critical illness.

A form of dialytic therapy known as continuous renal replacement therapy is used for approximately 5% of critically ill patients who are hemodynamically compromised and require the removal of fluid, toxic waste, and electrolytes but at a more regulated volume continuously over time.^13^ Free carnitine is of small molecular size and easily filtered with the dialysis process.^14^ Carnitine deficiency is commonly related to dialysis losses,^2^ and the incidence increases with the duration of continuous renal replacement therapy.^2^

During critical illness, the amount of carnitine consumed or provided in parenteral nutrition may be insufficient to maintain carnitine stores, contributing to a deficient state.^15^ Under normal conditions, when the body is not in a state of catabolism, carnitine can be derived from the breakdown of lysine and methionine.^16^ Carnitine is not routinely added to the adult parenteral nutrition prescription,^16^ and deficiency has been observed in the critically ill after prolonged enteral or parenteral nutrition support, either because of inadequate carnitine dosing in enteral nutrition or no carnitine added to the adult parenteral nutrition prescription.^16^ Carnitine deficiency should be considered with parenteral nutrition use of >2 weeks, particularly in the presence of elevated lactate or triglyceride levels.^17^

Medications such as valproic acid, which use carnitine as a transporter for excretion, increase carnitine losses.^18^, ^19^ This medication may also influence how much can be reabsorbed or endogenously produced.^15^ Other drugs that may have a similar impact on carnitine levels include pivampicillin and verapamil.^20^

This study aims to bridge a gap in the literature by identifying the prevalence of carnitine deficiency among a sample of critically ill adult patients admitted to either a surgical or cardiovascular intensive care unit, for whom carnitine data were collected during their intensive care stay. Knowing the prevalence of carnitine deficiency in critically ill patients is essential to determine whether this condition is an underrecognized contributor to critical illness. In addition to evaluating the presence of risk factors already potentially identified, this study also aimed to identify any other potential demographic and clinical predictors of carnitine deficiency, focusing on free carnitine levels as the sole indicator of deficient states. Understanding the prevalence of carnitine deficiency and identifying potential predictors of deficiency may inform further investigation in larger, more robust trials.

## METHODS

We performed a descriptive cross‐sectional study in a convenience sample of 144 critically ill adults admitted to a surgical or cardiovascular intensive care unit at the University of Utah Hospital who had serum carnitine levels measured from January 1, 2022, and December 31, 2024. The only exclusion criterion for those with carnitine levels measured was if they remained hospitalized at the time of data collection. Data were collected from Electronic Portfolio of International Credentials, the electronic health record (EPIC Systems Corporation, Verona, WI).

Variables describing the patient population, such as age, sex, body mass index, admission to surgical or cardiovascular intensive care unit, comorbidities, Acute Physiology and Chronic Health Evaluation II (APACHE II) score, intensive care length of stay, hospital length of stay, and therapies including continuous renal replacement therapy, parenteral nutrition, and valproic acid were included. Our primary outcome was carnitine deficiency defined as serum‐free carnitine level <36 μmol/L^21^ measured during the intensive care unit stay. Serum carnitine levels were measured using standardized hospital laboratory draws. The first available carnitine level closest to intensive care unit admission was used. Our secondary outcome was the acyl‐carnitine to free carnitine ratio determined on the same serum sample as the primary outcome and was calculated by dividing the esterified carnitine by the amount of free carnitine. The acyl‐carnitine to free carnitine ratio was reported for descriptive purposes of the sample. Because the ratio may be altered by factors unrelated to carnitine deficiency,^20^ we did not perform analyses examining predictors of the ratio.

Laboratory values that may be associated with carnitine deficiency, including elevations in liver enzymes such as alkaline phosphatase, aspartate aminotransferase, alanine aminotransferase, and total bilirubin, triglyceride, lactate, and ammonia, were reported as the levels measured closest to the timing of the carnitine testing. Each value was dichotomized using a yes or no classification for elevated levels, based on reference ranges from the literature.^22^, ^23^, ^24^, ^25^ Any point‐of‐care fingerstick blood glucose levels <60 mg/dl the week before carnitine testing were collected and dichotomized (yes/no, <60 mg/dl present).

Our study was approved by the ethics committees of the University of Utah Hospital and Rutgers University Institutional Review Boards (00173208 and study identifier Pro2024002279, respectively), adhering to the Declaration of Helsinki principles. Written informed consent was waived because the research posed no more than minimal risk to participants.

### Statistical analysis

For continuous variables in which data were normally distributed, mean and standard deviation were reported, and if data were not normally distributed, the median and interquartile range (IQR) were presented. For categorical variables, frequency distributions (*n*, %) were reported.

Bivariate analyses explored the relationships between all independent variables and the dependent dichotomous variable of carnitine status. As part of this analysis, chi‐square tests were used to examine the associations between categorical variables. The independent samples *t* test or Mann‐Whitney *U* test were used to evaluate associations between carnitine status and each continuous variable. A separate binary logistic regression model was constructed for each independent variable to identify predictors of carnitine deficiency, exploring potential predictors of carnitine deficiency based on the demographic and clinical characteristics of the sample. A multivariable regression model with carnitine deficiency as the outcome was then conducted by including age, sex, body mass index, a continuous renal replacement therapy prescription, a parenteral nutrition prescription, and a history of chronic kidney disease as independent variables. Multicollinearity was assessed for these independent variables by conducting correlation tests. A correlation coefficient ≥0.8 was used to indicate multicollinearity, and no multicollinearity was found among the independent variables included in the multivariable model. Results from the logistic regression analyses were presented as odds ratios (ORs) and 95% confidence intervals (CIs), with corresponding *P* values. Outliers were identified but not excluded from the analysis because they were determined to be biologically plausible and not impacting the results materially. The statistical analyses were performed with SPSS version 31.0 (IBM Inc, Armonk, NY), and significance testing was performed with the use of a two‐sided alpha level of 0.05. An a priori power analysis was conducted based on four potential predictors, including sex, body mass index, a prescription for continuous renal replacement therapy, and parenteral nutrition therapy prescribed. Assuming a medium effect size of 0.15 and an alpha level of 0.05, a sample size of approximately 250 would have been required for the study to be sufficiently powered at 80%, using the formula proposed for logistic regression modeling by Bujang et al.^26^

## RESULTS

### Patient population characteristics

Of the 144 patients identified with carnitine data available, the sample mean age was 56.5 (SD, 15.2) years, and 52.8% were men (Table 1). The mean body mass index, which is calculated as weight in kilograms divided by height in meters squared, was 30.3 (SD, 8.6), and the most common comorbidity was heart failure (35.5%), followed by diabetes mellitus (33.3%). The admitting intensive care unit was predominantly the surgical intensive care unit (69.4%). Nearly half of the sample received continuous renal replacement therapy at some point during their intensive care unit stay (45.8%), and 54.9% were prescribed parenteral nutrition. Over half (59.7%) of the sample were prescribed enteral nutrition at the time of carnitine testing. The mortality rate of 31.3% at the time of hospital discharge reflects the severity of illness of the sample population, coming from a high acuity level 1 trauma center.

**Table 1:** Demographic and clinical characteristics of the sample (*n* = 144).

Variable	Data
Age, mean (SD), years	56.5 (15.2)
Men, *n* (%)	76 (52.8)
BMI, mean (SD)	30.3 (8.6)
APACHE II score (*n* = 137), mean (SD)	18.7 (7.8)
Admitting ICU, *n* (%)	
Surgical ICU	100 (69.4)
Cardiovascular ICU	44 (30.6)
Comorbidity (*n* = 141), *n* (%)	
Heart failure	50 (35.5)
Diabetes mellitus	47 (33.3)
Hyperlipidemia	37 (26.2)
Chronic kidney disease	28 (19.9)
CRRT therapy prescribed, *n* (%)	66 (45.8)
Total CRRT days, median (IQR)	23.0 (7.8–35.0)
Number of CRRT days at the time of carnitine testing (*n* = 65), median (IQR)	11.0 (5.0–19.0)
ECMO therapy prescribed, *n* (%)	24 (16.7)
EN prescribed at goal at time of carnitine testing, *n* (%)	86 (59.7)
Length of hospital stay, median (IQR), days	30.5 (18.0–51.8)
Number of hospital days at time of carnitine testing, median (IQR)	14.5 (7.0–24.8)
Length of ICU stay (*n* = 137), median (IQR), days	17.1 (8.0–36.5)
Mortality at time of hospital discharge, *n* (%)	45 (31.3)
PN therapy prescribed, *n* (%)	79 (54.9)
Total PN days, median (IQR)	8.0 (5.0–14.0)
Number of PN days at the time of carnitine testing, median (IQR)	3.0 (0.0–7.0)
Valproic acid prescribed, *n* (%)	24 (16.7)
Total valproic acid days, median (IQR)	3.0 (2.0–3.8)
Number of days on valproic acid at time of carnitine testing, median (IQR)	1.0 (0.0–2.8)

Abbreviations: APACHE II, Acute Physiology and Chronic Health Evaluation II; BMI, body mass index; CRRT, continuous renal replacement therapy; ECMO, extracorporeal membrane oxygenation; EN, enteral therapy; ICU, intensive care unit; IQR, interquartile range; PN, parenteral nutrition.

### Prevalence of carnitine deficiency in the critically ill

Of the sample, 41.7% had carnitine deficiency (as defined by free carnitine levels <36 µmol/L), 46.5% were within the normal reference range of 36–74 µmol/L, and 11.8% had elevated levels >74 µmol/L (Table 2) with a median number of hospital days at time of carnitine laboratory testing of 14.5 (IQR, 7.0–24.8) (Table 1). An elevated acyl‐carnitine to free carnitine ratio of >0.4 was observed in 60.4% of the sample, with only 31.9% having a free carnitine <36 µmol/L and an acyl‐carnitine to free carnitine ratio >0.4 (Table 2). Those with an elevated acyl‐carnitine to free carnitine ratio >0.4 were more likely to be carnitine deficient than those with an acyl‐carnitine to free carnitine ratio ≤0.4. Abnormal laboratory values, including liver enzymes, were not significantly associated with carnitine deficiency for the sample.

**Table 2:** Select laboratory measurements of the sample (*n* = 144).

Variable	*n* (%)
Laboratory measurements related to carnitine status	
Free carnitine	
<36 µmol/L	60 (41.7)
36–74 µmol/L	67 (46.5)
>74 µmol/L	17 (11.8)
E/F ratio >0.4	87 (60.4)
Both free carnitine <36 µmol/L and E/F ratio >0.4	46 (31.9)
Other laboratory measurements that may be related to carnitine status^a^	
Alkaline phosphatase >129 U/L (*n* = 142)	59 (41.5)
AST >48 U/L (*n* = 142)	63 (44.4)
ALT >55 U/L (*n* = 142)	41 (28.9)
Total bilirubin >1.2 mg/dl (*n* = 142)	51 (35.9)
Serum triglyceride >150 mg/dl (*n* = 106)	53 (50.0)
Serum lactate >2 mmol/L	87 (60.4)
Serum ammonia ≥44 umol/L (*n* = 54)	19 (35.2)
Blood glucose ≤60 mg/dl	8 (5.6)

Abbreviations: ALT, alanine aminotransferase; AST, aspartate aminotransferase; E/F ratio, esterified to free carnitine ratio.

^a^ Refer to Methods section for citations related to reference ranges.

### Predictors of carnitine deficiency in the critically ill

There was a significant association between sex and carnitine status. Women were more likely to be carnitine deficient compared with men (52.9% vs 31.6%, respectively; *P* = 0.01). There was also a significant association between baseline diagnosis of chronic kidney disease and carnitine status, with 64.3% of those with chronic kidney disease being carnitine deficient compared with 36.3% of those without chronic kidney disease (*P* = 0.01). Those on continuous renal replacement therapy were more likely to be carnitine deficient than those not on continuous renal replacement therapy (54.5% vs 30.8%, respectively; *P* = 0.00) (Table 3). The mean body mass index was significantly lower among those who were carnitine deficient than for those who were not (28.5 vs 31.7, respectively; *P* = 0.03) (Table 4).

**Table 3:** Association between demographic or clinical characteristics of the sample and carnitine status.

Variable	Group	Carnitine Status, *n* (%)		*P* value^a^
		Free carnitine <36 µmol/L (*n* = 60, 41.7%)	Free carnitine ≥36 µmol/L (*n* = 84, 58.3%)	
Sex	Male	24 (40.0)	52 (61.9)	0.01
	Female	36 (60.0)	32 (38.1)	
Admitting ICU	CVICU	23 (38.3)	21 (25.0)	0.09
	SICU	37 (61.7)	63 (75.0)	
Diabetes mellitus	Yes	23 (39.0)	24 (29.3)	0.23
	No	36 (61.0)	58 (70.7)	
Heart failure	Yes	24 (40.7)	26 (31.7)	0.27
	No	35 (59.3)	56 (68.3)	
Hyperlipidemia	Yes	19 (32.2)	18 (22.0)	0.17
	No	40 (67.8)	64 (78.0)	
Chronic kidney disease	Yes	18 (30.5)	10 (12.2)	<0.01
	No	41 (69.5)	72 (87.8)	
Mortality at time of hospital discharge	Yes	22 (36.7)	23 (27.4)	0.24
	No	38 (63.3)	61 (72.6)	
EN prescribed at goal at the time of carnitine testing	Yes	37 (61.7)	49 (58.3)	0.69
	No	23 (38.3)	35 (41.7)	
Parenteral nutrition prescribed	Yes	30 (50.0)	49 (58.3)	0.32
	No	30 (50.0)	35 (41.7)	
CRRT prescribed	Yes	36 (60.0)	30 (35.7)	<0.01
	No	24 (40.0)	54 (64.3)	
Valproic acid prescribed	Yes	11 (18.3)	13 (15.5)	0.65
	No	49 (81.7)	71 (84.5)	

Abbreviations: CRRT, continuous renal replacement therapy; CVICU, cardiovascular intensive care unit, EN, enteral nutrition; ICU, intensive care unit; SICU, surgical intensive care unit.

^a^ Derived from Pearson chi‐square test.

**Table 4:** Association between key clinical variables and carnitine status (*n* = 144).

Variables	*n*	Free carnitine <36 µmol/L	*N*	Free carnitine ≥36 µmol/L	*P* value
Age, mean (SD), years	60	56.6 (15.4)	84	56.5 (15.1)	0.99^a^
BMI, mean (SD)	60	28.5 (7.3)	84	31.7 (9.2)	0.03^a^
APACHE II score (*n* = 137), mean (SD)	58	19.1 (8.3)	79	18.4 (7.5)	0.62^a^
Number of days at the time of carnitine testing					
Hospital days, median (IQR)	60	16.0 (5.0–25.3)	84	14.0 (8.0–24.8)	0.68^b^
PN days (*n* = 79), median (IQR)	30	1.5 (0.0–5.0)	49	4.0 (0.0–8.0)	0.12^b^
CRRT days (*n* = 65), median (IQR)	36	14.0 (5.5–20.8)	29	8.0 (4.5–14.0)	0.06^b^
Valproic acid days (*n* = 24), median (IQR)	11	1.0 (0.0–2.0)	13	2.0 (0.5–3.0)	0.25^b^

Abbreviations: APACHE II, Acute Physiology and Chronic Health Evaluation II; BMI, body mass index; CRRT, continuous renal replacement therapy; IQR, interquartile range; PN, parenteral nutrition.

^a^ *P* values are derived from the independent samples *t* test.

^b^ *P* values are derived from the Mann‐Whitney *U* test.

In the univariable binary logistic regression model analyzing carnitine status and demographic and clinical characteristics, women were 2.44 times more likely to exhibit carnitine deficiency than men (OR, 2.44; 95% CI, 1.24–4.81; *P* = 0.01) (Table 5). For each unit decrease in body mass index, the odds of developing carnitine deficiency increased by 5% compared with maintaining normal free carnitine status (OR, 0.95; 95% CI, 0.91–1.00; *P* = 0.03). Those prescribed continuous renal replacement therapy were 2.7 times more likely to develop carnitine deficiency than those not on continuous renal replacement therapy (OR, 2.70; 95% CI, 1.36–5.34; *P* = 0.00), and a history of chronic kidney disease was associated with a 3.16 times higher likelihood of carnitine deficiency compared with those without chronic kidney disease (OR, 3.16; 95% CI, 1.33–7.49; *P* = 0.00). Age and being prescribed parenteral nutrition therapy were not significant predictors of carnitine deficiency. Likewise, the sample's other demographic and clinical characteristics, including APACHE II score and comorbidities, were not significant predictors of carnitine status. When proceeding further with the multivariable model including age, sex, body mass index, a prescription for continuous renal replacement therapy, a prescription for parenteral nutrition, and chronic kidney disease, the association between sex and carnitine deficiency was of borderline statistical significance (OR, 2.07; 95% CI, 0.99–4.32; *P* = 0.05). Body mass index remained a significant predictor (*P* = 0.03). For those prescribed continuous renal replacement therapy, the observed association was slightly attenuated when findings were adjusted for age, sex, body mass index, parenteral nutrition therapy prescribed, and history of chronic kidney disease (OR, 2.43; 95% CI, 1.12–5.25; *P* = 0.02), but a history of chronic kidney disease was no longer associated with carnitine deficiency compared with those without chronic kidney disease (OR, 2.18; 95% CI, 0.84–5.63; *P* = 0.11). We conducted a sensitivity analysis by excluding outliers for hospital length of stay, duration of continuous renal replacement therapy, and parenteral nutrition therapies at the time of carnitine testing. Because removing outliers did not significantly change the results, the original dataset, which included outliers, was used for all data analyses.

**Table 5:** Potential predictors of carnitine deficiency.

Potential predictor	Univariable model			Multivariable model^a^		
	OR	95% CI	*P* value	OR	95% CI	*P* value
Age	1.00	0.98–1.02	0.99	1.00	0.98–1.03	0.98
Sex, female	2.44	1.24–4.81	0.01	2.07	0.99–4.32	0.05
BMI	0.95	0.91–1.00	0.03	0.95	0.90–0.99	0.03
CRRT prescribed, yes	2.70	1.36–5.34	<0.01	2.43	1.12–5.25	0.02
PN therapy prescribed, yes	0.71	0.37–1.39	0.32	0.79	0.37–1.70	0.55
History of CKD, yes	3.16	1.33–7.49	<0.01	2.18	0.84–5.63	0.11

*Note*: Reference category for carnitine deficiency is a normal free carnitine status. *N* = 144, except history of CKD (*n* = 141).

Abbreviations: BMI, body mass index; CI, confidence interval; CKD, chronic kidney disease; CRRT, continuous renal replacement therapy; OR, odds ratio; PN, parenteral nutrition.

^a^ Multivariable model included age, sex, BMI, CRRT prescribed, PN therapy prescribed, and CKD diagnosis (*n* = 141).

## DISCUSSION

Carnitine deficiency was observed in a significant number of critically ill patients. In the present descriptive study, free carnitine levels <36 µmol/L, suggestive of carnitine deficiency, were detected in over 40% of the patients. We observed a higher prevalence of carnitine deficiency than other published studies,^20^ which may have been influenced by our clinical judgment in those selected for carnitine testing because of suspicion for possible carnitine deficiency. In contrast, other studies looked at carnitine levels earlier, such as at the time of admission to the intensive care unit, not allowing the duration of critical illness to be considered.^20^ Furthermore, the free carnitine level at which deficiency is defined can vary by treatment facility and reference range used, with some cutoff ranges at <20 µmol/L,^16^ which would capture fewer patients. Across published studies,^4^, ^6^, ^13^, ^16^, ^20^, ^27^ various laboratory values are used to determine carnitine deficiency, including free carnitine, total carnitine, and the acyl‐to‐free carnitine ratio, leading to potential discrepancies in the number of carnitine‐deficient cases identified. In the present study, the acyl‐carnitine to free carnitine ratio identified more patients deficient than the free carnitine levels, which aligns with an acyl‐carnitine to free carnitine ratio >0.4 representing causes not directly related to a need for more carnitine, including impaired acyl‐carnitine excretion.^20^ As such, the acyl‐carnitine to free carnitine ratio may be referred to as a relatively deficient state^28^ vs absolute or an insufficient state rather than a deficient state.^29^ For clinical practice, this may infer that an elevated acyl‐carnitine to free carnitine ratio may be influenced by factors other than carnitine deficiency. However, when supported by a coexisting low free carnitine level, it can justify the need for supplementation.

We did not observe significant associations between liver enzymes, lactate, or triglyceride levels related to carnitine status. However, we may not have seen as many prolonged deficiency states as observed in the literature, in which carnitine deficiency worsened over time in the setting of proposed risk factors such as weeks on continuous renal replacement therapy, parenteral nutrition therapy, or the length of hospital stay.^16^ The European Society for Clinical Nutrition and Metabolism (ESPEN) advises that the combination of elevated triglycerides and lactate in the setting of a high degree of muscle wasting and fatty liver may indicate complications of carnitine deficiency.^11^ Inadequate carnitine may contribute to elevated ammonia levels with limited beta‐oxidation.^4^ Inadequate carnitine may result in inadequate fuel for the body, and hypoglycemia has been known to occur.^6^ In facilities where carnitine laboratory values are not readily accessible, being aware of other laboratory value abnormalities that may result from carnitine deficiency can help justify carnitine supplementation.

In the univariable model, a prescription for continuous renal replacement therapy, a history of chronic kidney disease, female sex, and a lower body mass index were significant predictors of carnitine deficiency in our study. Multivariable model results sustained as significant predictors of carnitine deficiency, a prescription for continuous renal replacement therapy, and a lower body mass index, but a history of chronic kidney disease was no longer significant; because statistical significance was defined as *P* < 0.05, results for female sex with *P* = 0.05 were considered marginal. Ostermann et al measured effluent losses and detected that carnitine was removed, confirming that carnitine is lost with continuous renal replacement therapy.^27^ Sgambat and Moudgil observed that one‐third of their sample was carnitine deficient before initiating continuous renal replacement therapy, and total and free carnitine levels progressively declined with the duration of continuous renal replacement therapy, resulting in all patients being deficient by week 3 of continuous renal replacement therapy.^6^ In a retrospective chart review, Fah et al observed that 70.6% of those on continuous renal replacement therapy were carnitine deficient compared with only 15.8% of those not requiring continuous renal replacement therapy (*P* < 0.001).^13^ Our numbers for those with carnitine deficiency were fewer in the continuous renal replacement therapy group in comparison. However, our median duration of therapy was shorter at 14 days, and carnitine measurements may have taken place before or after continuous renal replacement therapy therapy was initiated. The fact that carnitine deficiency occurs in those who are not prescribed continuous renal replacement therapy draws attention to the need to explore other potential predictors.

In the present study, chronic kidney disease was a statistically significant predictor of carnitine deficiency in the univariable model but not sustained in the multivariable model. In patients with chronic kidney disease who are dialysis dependent at baseline, inadequate muscle strength and fatigue have been known to trigger carnitine supplementation.^10^ Given that the kidney is primarily responsible for the resorption of carnitine to maintain serum carnitine levels,^1^ baseline kidney dysfunction appears logical as a potential predictor and warrants additional research.

Our results also showed that carnitine deficiency was more likely to occur in those of a lower body mass index, similar to observations by Oami et al, who found that body mass index was significantly lower in the carnitine‐deficient group (19.4 ± 2.9) compared with the group with normal carnitine (22.4 ± 2.8; *P* = 0.01).^20^ Although not a direct indicator of reduced muscle mass, a lower body mass index may be a marker of a subpopulation at greater risk for carnitine deficiency related to a decreased muscle reserve.^30^ Our finding that women were more likely than men to be carnitine deficient aligns with studies of reduced muscle mass observed in the female population compared with men.^31^

We did not observe a significant difference in the APACHE II score by carnitine status, which may be related to our sample having a similar level of illness acuity and limited score variability. Oami et al observed that higher Sequential Organ Failure Assessment scores, a marker of disease severity and organ failure, were associated with downward trends over time in carnitine levels. Likewise, they found no significant difference in APACHE II scores between the carnitine‐sufficient and carnitine‐deficient groups.^20^

Our study had several limitations. First, any inferences reported in this study are descriptive and should be interpreted as exploratory with the goal of inspiring future analytical studies. In addition, we cannot completely rule out the possibility of residual confounding. This was a single‐center study, limited to those with carnitine measures available, which may have reflected a higher incidence of carnitine deficiency if laboratory measures were checked in the setting of suspected deficient states. We could not control the timing of carnitine testing, making it challenging to capture whether the duration of therapies was a predictor of carnitine status. Other limitations of this study are the small number of patients on medications such as valproic acid and inadequate data on nutrition feeding volumes achieved to determine if the amount of nutrition support therapy provided might also be important to carnitine status. Checking carnitine status was a new practice implemented in our intensive care units that limited our numbers. Although not statistically powered, this is, to date, the largest known study investigating carnitine status in critically ill patients (*n* = 144). Current studies assessing predictors of carnitine deficiency range from 42–106 patients.^6^, ^13^, ^20^, ^27^, ^32^, ^33^ Variables not found to be significant in this study warrant further exploration in a larger study with sufficient statistical power. Studies with longitudinal monitoring of carnitine levels with standardized testing intervals would clarify the impact of therapy duration on carnitine status.

## CONCLUSION

Carnitine deficiency was present in 41.7% of those tested for carnitine deficiency within our intensive care unit population. We explored predictors of carnitine deficiency and found that receiving continuous renal replacement therapy and a lower body mass index were significant predictors in the multivariable model, but that female sex and history of chronic kidney disease may warrant further exploration based upon significance in the univariable analysis. Given that carnitine deficiency was observed in this study, it is recommended that critically ill patients be routinely monitored for the development of carnitine deficiency. Studies on carnitine dosing within the critically ill population are needed to establish guidelines for standardizing supplementation. Larger studies with longitudinal data are needed to confirm and explore further carnitine deficiency predictors and whether carnitine supplementation can improve clinical outcomes.

## AUTHOR CONTRIBUTIONS

**Jennifer Gordon**: conceptualization, investigation, writing—original draft, methodology, writing—review and editing, formal analysis, and data curation. **Rebecca Brody**: writing—review and editing. **Hamed Samavat**: formal analysis and writing—review and editing. **Mireille Hamdan**: writing—review and editing. **Alexander L. Colonna**: writing—review and editing. **Jason B. Young**: writing—review and editing. **Laura D. Byham‐Gray**: conceptualization and writing—review and editing.

## CONFLICT OF INTEREST STATEMENT

None declared.
